# Food insecurity profile according to habits and lifestyles in workers with subsistence jobs, Medellín-Colombia 2016

**DOI:** 10.47626/1679-4435-2022-1032

**Published:** 2023-11-24

**Authors:** María Osley Garzón-Duque, Fabio Leon Rodriguez-Ospina, Mariana Butinof, Maria Doris Cardona-Arango, Ángela María Segura-Cardona, Jose Manuel Miranda-Muñoz

**Affiliations:** 1 Universidad CES, Facultad de Medicina, Medellín, Antioquia, Colombia; 2 Universidad de Antioquia, Facultad Nacional de Salud Pública, Medellín, Antioquia, Colombia; 3 Universidad Nacional de Córdoba, Facultad de Ciencias Médicas, Córdoba, Argentina; 4 Universidad CES, Escuela de Graduados-Facultad de Medicina, Medellín, Antioquia, Colombia; 5 Universidad CES, Escuela de Graduados, Medellín, Antioquia, Colombia

**Keywords:** working conditions, food safety, environmental health, public health, epidemiology, condiciones de trabajo, seguridad alimentaria, salud ambiental, salud pública, epidemiología

## Abstract

**Introduction:**

Although the informal economy absorbs a considerable portion of the population, there is still little evidence that contributes to identify the lifestyles and eating habits that outline food insecurity in workers with subsistence jobs.

**Objectives:**

To determine the profile of food insecurity according to habits and lifestyles in workers with subsistence jobs.

**Methods:**

Cross-sectional study with primary sources of information, obtained through an assisted survey, to a census of 686 workers in 2016. The results of the nutritional component of a doctoral thesis approved by the Institutional Ethics Committee of the CES University, Medellín, Colombia, are presented.

**Results:**

In this working population, 52.6% were 50 years or old; however, 86.1% reported being the main household provider, and 33.3% did not have a permit to work in public areas. Moreover, 56.8% reported being sedentary/little active, and the highest consumption of alcohol and tobacco was recorded in men, who also ate their food alone. Conversely, women considered that mood affected their appetite, and they consumed their food while serving customers and handling money. In general, less than 50.0% of workers had a set time to consume their food. Food insecurity in workers’ households was explained by alcohol consumption (prevalence ratio = 1.62; confidence interval = 1.05;2.38) and having exclusive hours to consume food (prevalence ratio = 1.40; confidence interval = 1.00;1.96). Their food insecurity is defined by not consuming alcoholic beverages, considering that their mood affects their appetite, consuming one or two meals a day, without a defined schedule, not having permission to work, presenting moderate/severe food insecurity, and being a woman.

**Conclusions:**

The conditions that explain and outline food insecurity in this working population contribute to their socio-environmental and labor vulnerability, however, these conditions can be reversed.

## Abstract (es)

**Introducción:**

Aunque la economía informal ocupe una gran parte de la población, todavía existen pocas evidencias que ayuden a identificar los estilos de vida y hábitos alimentarios que definen la inseguridad alimentaria entre los trabajadores con empleos de subsistencia.

**Objetivos:**

Determinar el perfil de inseguridad alimentaria según hábitos y estilos de vida en trabajadores con empleos de subsistencia.

**Métodos:**

Estudio transversal con fuentes primarias de información, realizado a través de encuesta asistida, con un censo de 686 trabajadores en 2016. Los resultados del componente nutricional de una tesis doctoral aprobada por el Comité de Ética Institucional de la Universidad CES, en Medellín, Colombia, son presentados.

**Resultados:**

De esta población trabajadora, el 52,6% tenía más de 50 años, sin embargo, el 86,1% reportó ser la persona que más aportaba en el hogar y al 33,3% no se le permitía trabajar en los espacios públicos. El 56,8% refirió ser sedentario/poco activo, registrándose el mayor consumo de bebidas alcohólicas y cigarrillos en los hombres, quienes también comían solos. Por su parte, las mujeres consideraban que el estado de ánimo afectaba al apetito y, cuando comían, simultáneamente atendían a los clientes y manejaban dinero. En general, menos del 50,0% tenía un tiempo definido para consumir sus alimentos. Explicaron inseguridad alimentaria en sus hogares, consumo de bebidas alcohólicas (RP = 1,62. IC = 1,05;2,38) e horarios exclusivos para consumir alimentos (RP = 1,40. IC = 1,00;1,96). Perfilan su inseguridad alimentaria no consumir bebidas alcohólicas, considerar que el estado de ánimo afectaba al apetito, consumir una o dos comidas al día, sin horario definido, no tener permiso para trabajar, presentar inseguridad alimentaria moderada/grave y ser mujer.

**Conclusiones:**

Las condiciones que explican y perfilan la inseguridad alimentaria de esta población trabajadora contribuyen a configurar su vulnerabilidad socioambiental y laboral, sin embargo, pueden ser revertidas.

## INTRODUCTION

Economic adjustments have led to structural changes in developing countries,^1^ where the way of living and working has been altered by the reconfiguration of productive sectors, in which informal workers are compelled to take subsistence jobs,^2^ due to their low educational level, weak linkages to the formal economy, and difficulties in affiliating with the general social security system.^3^

These are conditions that may change the lifestyle and the eating habits in the working population absorbed by the informal economy, and sedentary lifestyle, consumption of foods rich in fat, flour, and sugar,^4,5^ and scarce time for eating have caused problems with unknown consequences, which could related to overweight and obesity.^5-8^ Although some sociodemographic, work, social security, and health characteristics of those working on the streets and sidewalks have been investigated,^5,7-13^ evidence is still scarce regarding their food insecurity and nutritional profile, which is partly determined by their way of working and the process of work overload and burnout^14^ that they experience in their everyday lives, considering the differences that may exist between men and women.

Prevalences of overweight and obesity above 30% have also been reported for informal workers,^7,8,12,15^ a concerning situation, given the complications derived from this condition, such as chronic and degenerative diseases that have been related with overweight and obesity,^16-18^ including diabetes^16^ and arterial hypertension,^19^ which, along with household food insecurity, turned obesity in poor populations into another public health problem.

Although the International Labor Organization (ILO) promotes heathy eating in the workplace,^1^ these policies apply more to workers in the formal sector,^2,5^ but, they are difficult to implement for those who have subsistence jobs, among other reasons because the immediacy of their struggle for daily survival, leading them to change their lifestyle and eating habits, which deteriorate their living and health conditions.

The evidence generated in this study may allow for facilitating the implementation of the occupational health and safety strategies for the informal working population, set forth in the Colombia’s 2012-2021 ten-year public health plan^20^ and in Medellín’s food security policy.^21^ Conversely, it is necessary to go beyond indicating the risk that informal workers could pose to public health, due the food they sell^10,11^ on the street, with the understanding that they are also at food and nutritional risk and can suffer from its consequences.

For the reasons give above, the present study aimed to determine the food insecurity profile of workers with subsistence jobs in the city of Medellín, in Colombia, for 2016, according to their eating and nutritional habits and lifestyle, in order to provide information that helps identify their socio-occupational and environmental vulnerability condition, bearing in mind the possible peculiarities of men and women, and to propose actions that allow for them to overcome this condition in local and national public policies for this type of working population.

## METHODS

*Design*: exploratory - cross-sectional with primary sources of information, derived from the doctoral thesis entitled “Vulnerabilidad sociolaboral y ambiental de un grupo de trabajadores informales “venteros” del centro de Medellín, bajo el modelo de Fuerzas Motrices. Medellín 2015-2019”.

*Population*: survey with 686 workers contacted by their leaders and by the primary investigator at their market stalls, at meetings, and at guild assemblies. A group of leaders and workers participated in study design and in the execution of field work, in the context of a process of joint construction and validation of methodologies and results. These data were obtained through an assisted survey at one of workers’ guild headquarters.

*Inclusion criteria*: >18 years old, with ≥5 years of professional experience, and who agreed to participate after being informed about the study, its procedures, benefits, scopes, and limitations. Workers who had left their job ≥1 year and who did not sign the written informed consent before data collected were excluded. The study was approved by the Institutional Ethics Committee - Universidad CES, Medellín. Record No. 84-code 470 of 2015.

*Variables.* Outcome dependent analysis: *food insecurity*, to screen for workers’ households at risk for food insecurity using the Latin American and Caribbean Food Security Scale (*Escala Latinoamericana y Caribeña de Seguridad Alimentaria y Nutricional*, ELCSA),^22^ which is composed of 15 yes/no questions, eight of them directed to the adults in the household in general, and seven specific for households with children and adolescents younger than 18 years of age. Cutoff points: a score of zero indicates food security; from 1 to 5, mild food insecurity; from 6 to 10, moderate food insecurity; and from 11 to 15 points, severe food insecurity. For analysis purposes, results were recategorized into: moderate/severe food insecurity (scores from 6 to 15) and food security/mild food insecurity (scores from zero to 5).

*Explanatory variables independents analysis. Demographic*: sex (workers’ biological condition), age recategorized into two groups (18 to 49, y ≥50 years), main household provider, marital status, and work permit. *Lifestyle and eating habits specific to their work*: physical activity level, consumption of tobacco and alcohol, use of saltshaker on the table, having a set time for consuming food, consumption of vitamin supplements, where the food was prepared (home, restaurant), with whom the worker usually eats, whether mood affects their appetite, duration of food storage at their workplace before consumption, number of daily meals, having exclusive hours to consume food, serving customers and handling bills and/or coins while eating, and cooking methods (baked, roasted, fried, steamed).

*Descriptive analysis:* descriptive, bivariate, and multivariate analysis to explore non-causal association between explanatory variables and food insecurity in workers’ households. The frequencies and percentages for all dependent variables and the independent (food insecurity: moderate/severe; security/mild insecurity) were calculated. Statistical association chi-square tests were performed to PR with 95% CI. A multivariate analysis with multiple logistic regression was conducted to identify the factors that explain moderate/severe food insecurity in workers’ households. Variables were added from the lowest to the highest p-value, based on the results for bivariate analysis and meeting the Hosmer-Lemeshow criterion (p < 0.25).

*Interdependent multivariate multiple correspondence analysis:* statistical technique appropriate for the identification of profiles, in order to identify group of categories or characteristics “profiles” of the variables analyzed. In this case, the technique was used to identify the profile of workers’ food insecurity, according to specific characteristics of their work, including variables that presented association and explanatory power in dependent bivariate and multivariate analyses, and their association was verified by the chi-square test. *Variables included*: sex, work permit, eating while serving clients and having exclusive hours to consume food, consumption of alcohol, mood affects appetite, having a set time to consume food, and number of daily meals. Furthermore, food insecurity was included as variable of location (to identify where the categories of moderate/severe food insecurity were located and their agreement or not with the most unfavorable conditions in workers).

Statistical tests were conducted with a confidence level of 95% and error of 5.0%. Calculations were made using SPSS^®^, version 21 (license of Universidad CES), Epidat version 3.1. Layout of tables and texts were made using Excel and Word software.

## RESULTS

### SOCIODEMOGRAPHIC CONDITIONS

Female workers had a lower age than their male counterparts. The greatest percentage of workers was aged from 45 to 59 years, of which 52.6% were >50 years old. Most participants were head of the family (86.1%) and had a partner (56.9%), a condition that was less prevalent in men. Furthermore, 33.3% (266) of workers did not have a work permit (Table 1).

**Table 1:** Descriptive statistics of sociodemographic conditions, lifestyles, eating habits, and food insecurity in workers. Medellín, 2016

Condition or characteristic	Sex				Total	
	Male		Female			
	n	%	n	%	n	%
Age: four groups (years)						
18 to 29	10	34.5	19	65.5	29	4.2
30 to 44	94	49.5	96	50.5	190	27.7
45 to 59	189	60.2	125	39.8	314	45.8
60 or older	102	66.7	51	33.3	153	22.3
						
Age: two groups (years) - for bivariate analysis						
18 to 49	170	52.3	155	47.7	325	47.4
50 or older	225	62.3	136	37.7	361	52.6
						
Main household provider						
Yes	343	57.9	249	42.1	592	86.3
No	52	55.3	42	44.7	94	13.7
						
Marital status						
Without a partner	179	60.5	117	39.5	296	43.1
With a partner	216	55.4	174	44.6	390	56.9
						
Permit to work in public areas						
Yes	141	53.0	125	**47.0**	266	38.8
No	254	60.5	166	39.5	420	61.2
						
Lifestyles - eating habits						
Physical activity level						
Sedentary	95	**24.0**	103	**35.5**	198	28.9
Little active	114	28.9	77	26.6	191	27.9
Active	127	32.2	68	23.4	195	28.5
Very active	59	14.9	42	14.5	101	14.7
Alcohol consumption						
Yes	134	**33.9**	41	**14.2**	175	25.6
No	261	66.1	248	85.8	509	74.4
Tobacco consumption						
Yes	79	**20.0**	39	**13.4**	118	17.2
No	316	80.0	251	86.6	567	82.8
Having a set time to consume food						
Yes	213	54.1	105	36.2	318	46.5
No	181	45.9	185	**63.8**	366	53.5
Consumption of vitamin supplements						
Yes	92	23.3	78	27.0	170	24.8
No	303	76.7	211	73.0	514	75.2
Use of saltshaker on the table						
Yes	95	24.1	67	23.1	162	**23.7**
No	299	75.9	223	76.9	522	76.3
Where food was prepared						
Home						
Yes	352	57.0	266	43.0	618	90.1
No	42	63.6	24	36.4	68	9.9
Restaurant						
Yes	274	63.6	157	36.4	431	63.1
No	120	47.4	133	52.6	253	36.9
Food is usually consumed						
Alone	230	80.0	132	45.5	45.4	362
Accompanied	165	20.0	158	54.5	54.6	323
Mood affects appetite						
Yes	116	29.4	129	**44.5**	245	35.8
No	279	70.6	162	161	55.5	440
Duration of food storage at market stall						
<1 hour	245	62.2	137	47.4	382	**55.9**
>1 h ≤3 hours	56	14.2	71	69	23.9	125
>3 h ≤5 hours	83	21.1	75	25.9	158	23.1
>5 hours	10	2.5	8	2.8	18	2.6
Number of daily meals						
One	18	4.6	29	10.0	47	6.9
Dos	132	33.4	123	42.4	255	37.2
Three	217	54.9	118	40.5	335	48.9
More than three	28	7.1	20	6.9	48	7.0
						
When consuming the main meals of the day						
The worker had exclusive hours for this activity						
Yes	147	37.3	77	26.6	224	32.7
No	247	62.7	213	73.4	460	**67.2**
The worker served customers						
Yes	244	61.9	213	73.4	457	**66.8**
No	150	38.1	77	26.6	227	33.2
The worker handled bills and/or coins						
Yes	232	58.9	200	69.0	432	**63.2**
No	162	41.1	90	31.0	252	36.8
						
Cooking methods						
Baked						
Yes	117	29.7	54	18.6	171	**25.0**
No	277	70.3	236	81.4	513	75.0
Roasted						
Yes	168	42.6	112	38.6	280	**40.9**
No	226	57.4	178	61.4	404	58.9
Fried						
Yes	137	34.8	85	29.3	222	**32.5**
No	257	65.2	205	70.7	462	67.5
Steamed						
Yes	135	34.3	81	27.9	216	**31.6**
No	259	65.7	209	72.1	468	68.4
						
Household food insecurity						
Moderate/severe	191	51.6	179	48.4	370	53.9
Security/mild insecurity	204	64.6	112	35.4	316	46.1

Content in bold: statistically significant association when p < 0.05.

### LIFESTYLES

Of the respondents, 56.8% (389) were sedentary or little active, a condition that was more prevalence in women (57.1%), who also presented higher rates of overweight/obesity (72.4%). Among the 15.6% (106) of workers with moderate/severe depression symptoms, there was a higher percentage of men (57.9%), who also exhibited higher rates of consumption of alcohol (33.9%) and tobacco (2.0%) (Table 1).

### EATING HABITS

Only 46.5% (318) of workers had a scheduled time for meals, a habit that was more common among men (54.1%). Less than 25.0% consumed vitamin supplements, and 23.7% (162) put a saltshaker on the table. More than 50.0% reported eating their meals alone, a habit that was particularly more frequent in men (80.0%). A higher percentage of women (44.3%) considered that mood affected their appetite. More than 25.0% of workers stored food ≥3 hours at their work stall before consumption. Moreover, 42.4% (123) of workers ate three meals daily; however, 52.4% (152) of women ate one or two meals (Table 1).

It was also observed that 32.7% of workers had exclusive hours to consume food; however, 66.8% (457) served customers while eating, and 63.2% (432) handled bills or coins; these habits were more prevalent in women. In general, respondents preferred roasted (40.9%) and fried (32.5%) foods, which were mainly prepared at home (90.1%) and at a restaurant (63.1%) (Table 1). Although moderate/severe food insecurity was present in 53.9% (370) of the households, women showed a prevalence of 61.5% for this phenomenon (179) (Table 1).

### SOCIODEMOGRAPHIC CONDITIONS AND LIFESTYLES ASSOCIATED WITH FOOD INSECURITY

The prevalence of food insecurity was significantly higher in women (prevalence ratio [PR] = 1.27; CI = 1.11;1.46) and in those who did not have a permit to work in public areas (PR = 1.95; CI = 1.42;2.67). Despite without statistical significance, the prevalence of food insecurity was higher in those aged from 18 to 49 years old (Table 2).

**Table 2:** Sociodemographic conditions and lifestyles associated with household food insecurity in workers participating in the study, Medellín 2016 (n = 686)

Characteristic -condition	Food insecurity		Total	Chi square (p value)	PR (95% CI)^*^
	Moderate/severe (n%)	Absent/ mild (n%)			
Sociodemographic conditions					
Sex					
Female	179 (61.5)	112 (38.5)	291	11.59 (0.001)	1.27 (1.11;1.46)
Male	191 (48.4)	204 (51.6)	395		1.0
Age group (years)					
18 to 49	97 (51.1)	93 (48.9)	190	0.87 (0.349)	1.17 (0.84;2.64)
50 or older	273 (55.5)	223 (45.5)	496		1.0
Main household provider					
Yes	326 (55.1)	266 (44.9)	592	2.21 (0.137)	0.72 (0.46;1.11)
No	44 (46.8)	50 (53.2)	94		1.0
Marital status					
Without a partner	162 (54.7)	134 (45.3)	296	0.13 (0.716)	0.95 (0.70;1.28)
With a partner	208 (53.3)	182 (46.7)	390		1.0
Work permit					
No	200 (47.6)	220 (52.4)	420	17.20 (0.000)	1.95 (1.42;2.67)
Yes	170 (69.3)	96 (36.1)	266		1.0
Lifestyles					
Physical activity					
Sedentary	110 (55.6)	88 (44.4)	198	1.51 (0.680)	0.82 (0.50;1.32)
Little active	99 (51.8)	92 (48.2)	192		0.95 (0.58;1.58)
Active	110 (56.4)	85 (43.6)	195		0.79 (0.49;1.28)
Very active	51 (50.5)	50 (49.5)	101		1.0
Alcohol consumption					
Yes	80 (45.7)	95 (54.3)	175	6.60 (0.010)	1.57 (1.11;2.22)
No	290 (57.0)	219 (43.0)	509		1.0
Tobacco consumption					
Yes	70 (59.3)	48 (40.7)	118	1.61 (0.204)	0.77 (0.51;1.15)
No	300 (52.9)	267 (47.1)	567		1.0

* Statistically significant association when p < 0.05. PR = prevalence ratio

In relation to lifestyles, statistically significant associations were observed only between food insecurity and consumption of alcohol, showing that food insecurity was 57.0% more prevalent among those who consumed alcohol (PR = 1.57; CI = 1.11;2.22) (Table 2).

### EATING HABITS ASSOCIATED WITH FOOD INSECURITY

The prevalence of food insecurity was significantly higher (p < 0.05) among those who did not have a set time to consume their food (PR = 1.90; CI = 1.40;2.58) and among those who had exclusive hours for this activity (PR = 1.42; CI = 1.03;1.96). Despite without significant association, a higher prevalence of food insecurity was observed among those who used vitamin supplements, who consumed food prepared at home, who stored food in their market stall for ≥3 hours before consumption, consumed food prepared at a restaurant, and ate preferably alone (Table 3).

**Table 3:** Eating habits associated with food insecurity among workers participating in the study. Medellín 2016 (n = 686)

Characteristic - condition	Food insecurity		Total	Chi-square (p value)	PR (95%CI)^*^
	Moderate/severe (n%)	Absent/ mild (n%)			
Eating habits					
Set time to consumed food					
Yes	145 (45.6)	173 (54.4)	318	17.12 (0.000)	1.90 (1.40;2.58)
No	225 (61.5)	141 (38.5)	366		1.0
Consumption of vitamin supplements					
Yes	82 (48.2)	88 (51.8)	168	3.11 (0.078)	1.39 (0.97;1.94)
No	288 (56.0)	226 (44.0)	514		1.0
Use of saltshaker at the table					
Yes	86 (53.1)	76 (46.9)	162	0.06 (0.806)	0.96 (0.67;1.36)
No	283 (54.2)	239 (45.8)	522		1.0
Where food was prepared					
Home					
Yes	328 (53.1)	290 (46.9)	618	2.65 (0.104)	1.55 (0.91;2.62)
No	42 (63.6)	24 (36.4)	68		1.0
Restaurant					
Yes	238 (55.2)	193 (46.9)	431	0.59 (0.440)	0.88 (0.65;1.21)
No	132 (52.2)	121 (47.8)	253		1.0
Where food is consumed					
At worker’s market stall					
Yes	295 (54.0)	251 (46.0)	546	0.00 (0.988)	1.00 (0.68;1.45)
No	75 (54.0)	64 (46.0)	139		1.0
At a restaurant					
Yes	71 (51.8)	66 (48.2)	137	0.33 (0.565)	1.12 (0.77;1.62)
No	299 (54.6)	249 (45.4)	548		1.0
With whom worker consumes food					
Alone	190 (52.5)	172 (47.5)	362	0.72 (0.396)	1.14 (0.84;1.54)
Accompanied	180 (55.7)	143 (44.3)	323		1.0
Mood affects appetite					
Yes	164 (66.9)	81 (33.1)	245	25.15 (0.000)	0.43 (0.31;0.60)
No	206 (46.8)	234 (53.2)	440		1.0
Duration of food storage at point of sale before consumption (hours)					
≥3	85 (48.3)	91 (51.7)	176	3.02 (0.082)	1.36 (0.96;1.91)
<3	285 (55.9)	225 (44.1)	510		1.0
Number of daily meals					
One	34 (72.3)	13 (27.7)	47		0.17 (0.07;0.42)
Two	168 (65.9)	87 (34.1)	255	38.77 (0.000)	0.23 (0.12;0.46)
Three	153 (45.7)	182 (54.3)	335		0.54 (0.28;1.03)
More than three	15 (31.3)	33 (68.8)	48		1.00
When eating					
The worker had exclusive hours for this activity					
Yes	108 (48.2)	116 (51.8)	224	4.62 (0.032)	1.42 (1.03;1.96)
No	262 (57.0)	198 (43.0)	458		1.0
The worker served customers simultaneously					
Yes	260 (56.9)	197 (43.1)	457	4.33 (0.037)	0.71 (0.52;0.98)
No	110 (48.5)	117 (51.5)	227		1.0
The worker handled bills or coins simultaneously					
Yes	242 (56.0)	190 (44.0)	432	1.74 (0.186)	0.81 (0.59;1.10)
No	128 (50.8)	124 (49.2)	252		1.0

* Statistically significant association when p < 0.05. PR = prevalence ratio.

Lower prevalences (p < 0.05) of food insecurity were found among those who considered that mood affected their appetite (PR = 0.43; CI = 0.31;0.60), who served customers while eating (PR = 0.71. CI = 0.52;0.98), and who had three daily meals or less (Table 3).

### LIFESTYLES AND EATING HABITS THAT EXPLAIN HOUSEHOLD FOOD INSECURITY

After adjustment for variables associated with food insecurity or that met the Hosmer-Lemeshow criterion (p < 0.25) by multiple logistic regression, alcohol consumption (PR_adjusted_ = 1.62; CI = 1.05;2.38) and having a set time to consume their food (PR_adjusted_ =1.40; CI = 1.00;1.96) were identified as the conditions that explain (p < 0.05) higher food insecurity in workers’ household. Despite without statistical significance, greater food insecurity could also be explained by being a man, consuming food prepared at home, storing food for ≥3 hours at worker’s market stall before consumption, having exclusive hours to consume food, using vitamin supplements, and handling bills or coins while eating (Table 4).

**Table 4:** Lifestyles and workers’ eating habits that explain food insecurity in their households. Medellín 2016

Condition - characteristic	Crude PR	95%CI		Adjusted PR	95%CI	
		LT	UT		LT	UT
Sex - Male	1.71	1.25	2.32	1.28	0.90	1.83
Main household provider - Yes	0.72	0.46	1.11	0.75	0.46	1.20
Work permit - No	1.95	1.42	2.67	0.58	0.41	0.82
Alcohol consumption - Yes	1.57	1.11	2.22	1.62	1.05	2.38
Tobacco consumption - Yes	0.77	0.51	1.15	0.79	0.50	1.24
Fixed time for eating - Yes	1.90	1.40	2.58	1.40	1.00	1.96
Use of vitamin supplements - Yes	1.39	0.97	1.94	1.13	0.78	1.65
Consumption of food prepared at home - Yes	1.55	0.91	2.62	1.46	0.81	2.64
Mood affects appetite - Yes	0.43	0.31	0.60	0.51	0.36	0.73
Duration of food storage at workers’ market stall before consumption - ≥3 hours	1.36	0.96	1.91	1.26	0.86	1.85
Number of daily meals - RC >3	1.00	-	-	1.00	-	-
One	0.17	0.07	0.42	0.28	0.11	0.72
Two	0.23	0.12	0.46	0.24	0.12	0.49
Three	0.54	0.28	1.03	0.45	0.23	0.88
When eating, the worker						
Takes exclusive time for this activity - Yes	1.42	1.03	1.96	1.85	0.50	6.82
Serves customers - Yes	0.71	0.52	0.98	0.73	0.17	3.20
Handles bills or coins - Yes	0.81	0.59	1.10	1.81	0.74	4.42

LT = lower threshold; PR = prevalence ratio; RC = reference criterion; UT = upper threshold.

Not having work permit was no longer a condition associated with higher food insecurity and started to explain lower food insecurity (PR_adjusted_ = 0.58. CI = 0.41;0.82). The other conditions maintained their direction of association from bivariate to multivariate analysis, as shown in Table 4.

### CONDITIONS THAT OUTLINE WORKERS’ FOOD INSECURITY AND NUTRITIONAL HABITS

**Figure 1:**
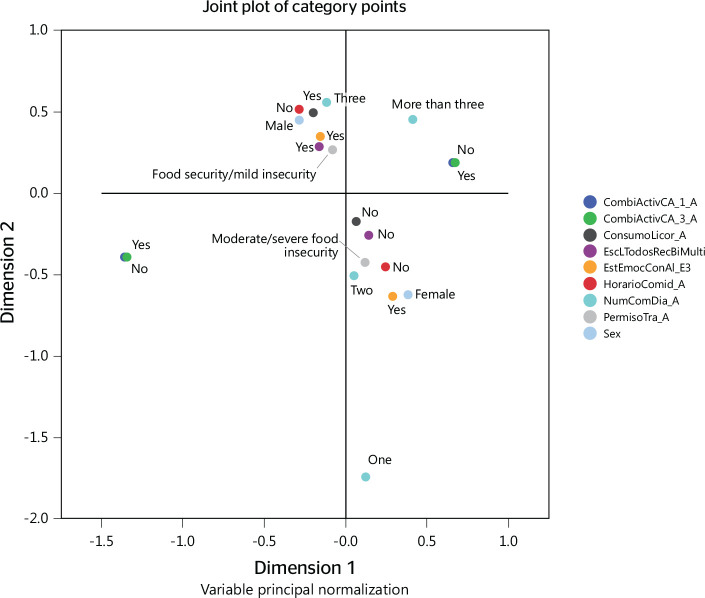
Profile of lifestyles and eating and nutritional habits in informal street vendors at the center of Medellín, Colombia, 2016.

As shown in Figure 1, the profile obtained resulted in three subgroups, with a total explained variance of 1.80, a Cronbach’s alpha of 0.587 (dimension 1) and of 0.408 (dimension 2), and mean Cronbach’s alpha of 0.510. The most representative discrimination variables were: consuming food while serving customers (0.895), having exclusive hours for eating (0.892), number of daily meals (0.471), and sex (0.110). The greatest correlation was observed for those who consumed food while serving customers and ate one daily meal.

The first quadrant of Figure 1 (dimension 2) included characteristics coinciding with the location of the variable, i.e., food security/mild insecurity: having a set time to consume food, consuming alcohol, eating three daily meals, mood affecting appetite, having a work permit, and being a man. The second quadrant (dimension 2) included workers who: ate more than three daily meals, did not serve customers while consuming food, and who had a set time to consume the main meals of the day (Figure 1).

The previous characteristics were opposed to that of the subgroup of workers belonging to the fourth quadrant (dimension 1), which includes women, individuals who did not consume alcohol, whose mood affected appetite, who ate one or two daily meals, who did not have a work permit, and who have a set time to consume food. Particularly, these characteristics coincide with the location of the supplemental variable “moderate/severe food insecurity” (Figure 1).

## DISCUSSION

Although there is evidence of a public health problem in Medellín, Colombia, this could reflect the conditions experienced by subsistence workers in other cities of the country and in other countries in Latin America and the Caribbean, regarding food insecurity faced by informal workers with subsistence job on the streets and sidewalks. This article includes part of the nutritional component addressed by the aforementioned doctoral thesis, in which food insecurity in workers’ households was screened by the ELCSA scale.^22^ However, frequency of consumption by type of food was not included in the analysis, which could also have contributed to understand the profile of workers’ vulnerability.

This study explored characteristics and factors little explored in this type of population, thus making it difficult to make detailed comparisons for each variable. Nonetheless, there are interesting elements to obtain knowledge on the issue and to allow for decision-making aimed to improve food security in the household of workers with subsistence jobs in Colombia and in other countries in the region as well.

### WORKERS’ SOCIODEMOGRAPHIC CONDITIONS

There was a higher proportion of workers aged from 45 to 59 years old, which indicated that, in a few years, they will be older adults with subsistence jobs, a situation that will make difficult for them to have save retirement guarantees, and despite the predominance of men, this situation may be changing, because, as reported by the OIT-2013 labor outlook, women are increasing more exposed to unemployment and job informality.^23^

Affiliation to the health insurance network was mainly via a subsidized scheme, similar to what was reported by other studies with informal workers^7,12,13,16,24^; affiliation to pension and occupational risk systems was practically nonexistent, in line with findings observed for potato farmers in Boyacá, Colombia.^8^ It was also observed that participants had low monthly incomes, despite being the main household provider, which is a noticeable factor that prevents them from overcoming their socio-occupational and environmental condition,^24^ due to the poverty in which they should survive.

### WORKERS’ LIFESTYLES AND EATING HABITS

In relation to lifestyles and eating habits, most workers were sedentary and little active, conditions that may lead to overweight and obesity,^15,17^, as previously reported for formal workers in the United States,^5,18^ Australia,^25^ Uruguay,^26^ Brazil,^27^ and Mexico,^28^ for sellers working in the Basurto market in Cartagena, Colombia,^7^ informal workers in Bucaramanga, Colombia,^16^ and in potato farmers in Boyacá.^8^ For the city of Medellín, where the workers evaluated in the present study lived, the prevalence of obesity in the general population^21,29^ was estimated to be higher for the more sedentary and physically inactive population, especially in the disadvantaged socioeconomic strata, to which most participants in this study belong.

It is important to highlight that sedentary lifestyle has been related to several health ailments, such as cardiovascular diseases, as shown in a study of workers in Spain,^30^ which reported that subjects who spent sitting from 4.8 to 4.6 hours daily had lower diastolic blood pressure than those who spent sitting for more than 6.6 hours daily, showing that, the higher the number of sedentary hours, the higher the prevalence of arterial hypertension.

Furthermore, one of every four workers consumed alcohol, a low consumption than that reported for potato farmers in Boyacá.^8^ Conversely, the prevalence of tobacco consumption was similar to that of these potato farmers,^9^ but lower to that found for workers from Plaza Minorista in Medellín.^19^

Most workers reported consuming their food at their market stall, a situation that could pose a risk for physical or mental disorders in both formal and informal workers; furthermore, these disorders could be aggravated by the fact of not having set hours to consume food, a recurrent situation among these workers and that was also observed by a study in Uruguay.^26^ These workers reported that consuming mainly one or two daily meals, especially lunch and dinner, a situation that could show that, although the frequency of consumption is low, the amount and the type of food consumed could partly explain the occurrence of overweight and obesity,^15^ whose prevalences are higher than those observed for Medellín’s general population.^29^

According to the descriptive note 311, issued in 2015 by the World Health Organization,^17^ “there has been an increase in the consumption of high-calorie foods rich in fat, salt, and sugar, and there has been a decrease in physical activity levels, partly as a consequence of sedentary lifestyle in many ways of working,” and these were some eating habits and lifestyles observed in the workers included in the present study. This fact makes it complex to implement food and nutritional security policies in Medellín and other cities worldwide, since, in part, these habits and lifestyles represent involuntary exposures for workers, who should accept that as part of the reality they live, where, more than being aware of what is good or bad for their health is the fact that they can really eat better and adopt good eating habits.

Before thinking of new pedagogic strategies that have an impact from the standpoint of health promotion and disease prevention, it is necessary to think of actions that help ensure them access to a safe and adequate diet and that also make it easier for them to adopt good habits and lifestyles.

### PROFILE OF FOOD AND NUTRITIONAL INSECURITY IN WORKERS’ FAMILIES

In Colombia, food security is related to sufficient and stable food availability, as well as access and timely and permanent consumption of food in the same amount, quality, and safety by everyone, under optimal conditions to lead to a healthy and active life, a situation that is also contemplated in the city of Medellín.^21^ However, this premise is difficult to implement in this working population, because their eating and nutritional habits facilitate and perpetuate food and nutritional insecurity in their households, whose prevalence at the time of the study was 53.9% (370) in general, 51.6% (191) for men and 48.4% (179) for women, who had the highest percentage of severe household food insecurity.

Finally, it is important to clarify that, despite no information was reported about some of the variables explored here for informal workers with subsistence jobs in Colombia and Latin America, nor how sociodemographic conditions, habits, and lifestyles are associated, explain, and outline moderate/severe food insecurity in informal workers’ families, a situation that limits the comparison of the findings observed here with those of other studies, it is necessary to report that, in this population, being a woman (PR_crude_ = 1.27. CI=1.11;1.46), not having a work permit (PR_crude_ = 1.95. CI=1.42;2.67), consuming alcohol (PR_crude_ = 1.57. CI = 1.11;2.22), having a set time to consume their food (PR_crude_ = 1.90. CI = 1.40;2.58), and having exclusive hours for this activity (PR_crude_ = 1.42. CI = 1.03;1.96) was associated with a higher prevalence of food insecurity. Conversely, its higher prevalence could be explained by alcohol consumption (PR_adjusted_ = 1.62. CI = 1.05;2.38) and having exclusive hours to consume food (PR_adjusted_ = 1.40. CI = 1.00;1.96).

A relevant evidence found in the present study was that the profile of food and nutritional insecurity of this working population reflects sex inequalities, since moderate/severe food insecurity have more noticeable characteristics in women, whose mood affected appetite, did not have a work permit, consumed food while serving customers and handling bills or coins, did not have a set time to consume food, and ate two daily meals or less, all situations that outline not only their food and nutritional vulnerability but also greatly contribute to their socio-occupational and environmental vulnerability and are in line with findings reported by the United Nations General Assembly regarding women’s vulnerability due to situations such as discrimination, violence, and gender inequality.

This information may facilitate the identification of the most relevant factors among social health determinants by sex, for the working population with subsistence jobs and who work on the streets and sidewalks, and may facilitate the search for resources and strategies that enable to solve or improve the issue, taking as the starting point the development of new studies and joint strategies with the government, workers, and their leaders to provide solutions at the short, medium, and long term, in order to address social and gender inequalities, to reduce household food insecurity, which could contribute to the presence of chronic, mental and degenerative diseases that could be prevented, and to improve the quality of life of the working population with subsistence jobs on the streets and sidewalks in Medellín and other cities in country and in the continent.
